# Skin fluorescence as a clinical tool for non-invasive assessment of advanced glycation and long-term complications of diabetes

**DOI:** 10.1007/s10719-016-9683-1

**Published:** 2016-06-10

**Authors:** Bernardina T. Fokkens, Andries J. Smit

**Affiliations:** 1 Department of Internal Medicine, University Medical Center Groningen (UMCG), Hanzeplein 1, 9713 GZ, Groningen, the Netherlands; 2 Research Institute GUIDE, Graduate School of Medical Sciences, University of Groningen, Antonius Deusinglaan 1, 9713 AV, Groningen, the Netherlands

**Keywords:** Skin autofluorescence, Skin intrinsic fluorescence, Advanced glycation endproducts, Diabetes mellitus, Diabetes complications

## Abstract

Glycation is important in the development of complications of diabetes mellitus and may have a central role in the well-described glycaemic memory effect in developing these complications. Skin fluorescence has emerged over the last decade as a non-invasive method for assessing accumulation of advanced glycation endproducts. Skin fluorescence is independently related to micro- and macrovascular complications in both type 1 and type 2 diabetes mellitus and is associated with mortality in type 2 diabetes. The relation between skin fluorescence and cardiovascular disease also extends to other conditions with increased tissue AGE levels, such as renal failure. Besides cardiovascular complications, skin fluorescence has been associated, more recently, with other prevalent conditions in diabetes, such as brain atrophy and depression. Furthermore, skin fluorescence is related to past long-term glycaemic control and clinical markers of cardiovascular disease. This review will discuss the technique of skin fluorescence, its validation as a marker of tissue AGE accumulation, and its use as a clinical tool for the prediction of long-term complications in diabetes mellitus.

## Introduction

Worldwide, the prevalence of diabetes mellitus is increasing at an alarming rate, mainly because of an increasing incidence of type 2 diabetes mellitus (T2DM) [1]. The concomitant rising medical costs are largely attributable to treatment of long-term complications caused by the glucose dysregulation. In order to prevent end-organ damage, such as cardiovascular complications, kidney failure, and cerebral complications, it is important to define involved pathophysiological mechanisms. Increased tissue advanced glycation endproducts (AGEs) play an important role in these mechanisms [2] and may be used as a biomarker of diabetes complications.

AGEs are formed in a multistep process by glycation and oxidation of free amino groups in proteins, lipids, and nucleic acids. These AGEs promote tissue dysfunction through cross-linking of long-lived molecules and through binding to the receptor for advanced glycation endproducts (RAGE) [3]. AGEs accumulate in the body during ageing, which results in structural and functional tissue impairment. Formation and accumulation of AGEs is accelerated in diabetes mellitus. Furthermore, increased accumulation of AGEs occurs in several other conditions, especially those associated with acute or chronic oxidative stress. In renal failure, tissue AGE levels are also increased due to impaired excretion of free AGEs and AGE peptides [4].

In 1986, Monnier *et al*. [5] first presented evidence that accumulation of AGEs in skin tissue was related to the presence of micro- and macrovascular complications in patients with type 1 diabetes mellitus (T1DM). Later, the DCCT-EDIC substudy on skin collagen glycation showed that dermal tissue AGE levels predict long-term diabetes complications in T1DM, even after adjustment for HbA1c [6]. The authors indicated that the damaging effect of AGEs in long-lived protein molecules, such as collagen, may explain the phenomenon that they called “metabolic memory”: the prolonged beneficial effects of intensive therapy and the deleterious effects of conventional, less stringent, therapy long after cessation of the relatively brief intervention period. In subsequent years, a range of experimental and clinical studies confirmed this relation of increased levels of AGEs, both in blood and slow-turnover tissues, with the presence of vascular damage and complications in both T1DM and T2DM. The United Kingdom prospective diabetes study (UKPDS) also proposed AGEs as likely candidates to explain the effect of metabolic memory [7].

However, assessment of AGEs in skin biopsy is not suitable for clinical use, because of the invasive method and high costs. In 2004, a first report appeared that skin fluorescence, non-invasively assessed, was related to AGE levels in dermal biopsies in diabetes patients and in healthy controls [8]. The dermal biopsies were obtained from the same measurement site as in which skin fluorescence was performed and several AGEs were determined using chromatographic and mass spectrometric methods. The study pointed out that skin fluorescence reflects dermal AGE levels and may be used as a biomarker in AGE-related diseases. This review will discuss the technique of skin fluorescence, its validation as a marker of tissue AGE accumulation, and its use as a clinical tool for the prediction of long-term complications in diabetes mellitus.

## Technique of skin fluorescence measurement

So far, two devices have been used for assessment of skin fluorescence in clinical studies: the AGE Reader (DiagnOptics technologies BV, Groningen, the Netherlands) and the SCOUT DS SF spectrometer (VeraLight, Inc., Albuquerque, NM, USA). Although both devices have been developed to reflect skin AGE accumulation, the measurement techniques differ, and the measurement values are not directly comparable. Therefore, skin fluorescence as measured by the AGE Reader, previously called Autofluorescence Reader (AFR), will be referred to as skin autofluorescence (SAF). Skin fluorescence as measured by the SCOUT device will be referred to as skin intrinsic fluorescence (SIF). When general statements on either device are made, the term ‘skin fluorescence’ will still be used.

SAF is measured on the volar side of the forearm. Care should be taken to perform this measurement in an area with normal skin with minimal sunlight exposure. The AGE Reader contains a UV-A light emitting lamp that emits light with a peak wavelength of 360–370 nm. Light reflected and emitted in the 300–600 nm range from the skin is measured in the research version by an inbuilt spectrometer, using a UV glass fibre. In later, simpler versions, the spectrometer is replaced by a set of photodiodes with peak sensitivities for different wavelengths. Before every measurement, dark and white reference readings are performed to correct for background light and to calculate reflectance, respectively. Initially, SAF measurements were not considered for analysis if the reflectance level was less than 10 %. After introduction of more sophisticated and validated skin colour correction software (version 2.3), this limit was lowered to 8 %. This adaptation allowed the use of SAF in a broader group of persons with dark skin colour. To correct for differences in light absorption, SAF is calculated as a ratio of excitation light (300–420 nm) to emitted light (420–600 nm). Consequently, SAF is expressed in arbitrary units (AU). Intra-observer variation of repeated autofluorescence measurements is 5 % to 6 % within one day in different publications [8].

Although the exact molecular structures and the diversity of species contributing to skin fluorescence are not established, tissue fluorescent species have their own specific excitation-emission spectrum. The used wavelength band of the AGE Reader was selected such that the major contribution in fluorescence comes from fluorescent AGEs. The majority of identified AGEs are characterized by fluorescence in the area around an excitation wavelength of 370 nm and an emission of 440 nm [9–11]. For example, the excitation spectrum of pentosidine is 335 nm and its emission spectrum 385 nm [12]. It was also investigated whether specific wavelengths should be preferred over the currently used wavelength band of the AGE Reader for differentiation between diabetic and non-diabetic subjects or between diabetic subjects with and without diabetes-related chronic complications. The authors concluded that their results showed the validity of applying a broad excitation wavelength range since no specific excitation or emission wavelengths would yield an increased distinction between the different groups [13].

SCOUT measurements are performed on the inner side of the forearm and are corrected for factors that affect light scattering and absorption as well. SIF is excited with a light-emitting diode (LED) centred at 375 nm, and the emission is detected over 435–655 nm (with the reflectance adjusted by the dimensionless excitation and emission exponents, kx = 0.6 and km = 0.2 respectively) [14]. In secondary analyses, SIF measurements were examined using excitation LEDs centred at 405 nm, 416 nm, 435 nm, and 456 nm. The VeraLight company, which developed the SCOUT device specifically for non-invasive diabetes detection, has suspended activities 2 years ago. Consequently, this device is no longer available.

## Validation studies

Several validation studies have been conducted to determine the performance of SAF as a tool for measuring dermal AGE accumulation. First, SAF was compared with several specific fluorescent and non-fluorescent AGEs in skin biopsies of diabetes patients and control subjects. Later, SAF was validated in a comparable way in renal failure and some other conditions. Moreover, SAF was compared to another classical AGE assay method for measuring AGEs, namely collagen-linked fluorescence [8, 15, 16]. A combined analysis of the validation studies of SAF against dermal tissue AGE levels was previously reported [17]. To illustrate, SAF had a correlation coefficient of 0.87 with dermal tissue pentosidine levels in biopsies from the same lower arm site, and so, SAF explained 76 % of the variance in dermal pentosidine levels [17].

SIF was originally based on a comparison of non-invasive fluorescence spectroscopy and skin collagen AGEs (determined using chromatographic and mass spectrometric methods) in a pig skin collagen model [18]. Further validation was performed in a human subject study in which measurements with an early SCOUT prototype were evaluated in diabetes *versus* control subjects. This study demonstrated that SAGE, a skin derived skin fluorescence parameter, could accurately classify disease in a case-control population [19]. However, SIF has not been validated in a direct comparison between skin fluorescence and dermal AGE levels in human subjects.

## Dark pigmentation, skin products, and other influencing factors

A remark on the use of skin fluorescence deserves attention; skin fluorescence measurements may be influenced by dark pigmentation, the use of skin products, the fasting state, and lifestyle factors.

For the AGE Reader, it has been demonstrated that SAF levels show expected relations with calendar age and presence of diabetes up to Fitzpatrick [20] skin phototype class IV. In contrast, SAF levels may not be reliable in dark skin types (Fitzpatrick class V-VI) [21, 22]. Therefore, ongoing studies with the AGE Reader address and aim to resolve the limitations in the use of SAF in people with dark skin types.

Several skin products may affect the measurement of SAF, especially sunscreens and skin tanners. However, some conventional skin creams may also make the measurement unreliable. When possible, persons should be asked to avoid use of skin products several days before a SAF measurement. Otherwise, persons should be asked about recent use of skin products [23].

An increase in SAF was seen 2 h postprandial in a study performed by Stirban *et al*. [24]. This may suggest that blood AGEs and redox-regulated fluorophores from skin micro vessels contribute to SAF in addition to skin-bound compounds. Therefore, it was suggested that performance of SAF in the fasting state might improve the sensitivity and specificity of the method. Although we should keep this in mind, the postprandial increase in SAF was limited (10 %) and not seen in all patients. Moreover, it is not clinically preferable to perform SAF in the fasting state, and most current knowledge concerning SAF, including its validation, has been acquired in patients irrespective of their fasting state. Furthermore, in a study where food intake was not restricted, the variation of SAF over the day was 5 % [25]. Therefore, it is not generally recommended to perform SAF after an overnight fast.

As AGEs accumulate over time, SAF increases with aging [21]. In addition, SAF may be influenced by other clinical and lifestyle factors, such as kidney function [26] and smoking [27]. Recently, an integrated analysis of both lifestyle and clinical factors has been performed in a large-scale non-diabetic population and a T2DM subpopulation [28]. The results show that 34 % of the variance in SAF was explained by age, body mass index, HbA1c, creatinine clearance, *N*-acetyltransferase 2 polymorphism, (pack-years of) smoking, and coffee consumption. In the T2DM population, 47 % of the variance in SAF was explained by the same factors except for coffee consumption.

For the SCOUT device, it was claimed that the skin colour range, in which reliable measurements could be made, was somewhat broader than for the AGE Reader. In subjects at risk for T2DM, it is observed that SIF measurements are not majorly influenced by the fasting status since the variation (5.7 %) for fasting subjects (pre-glucose challenge *versus* post-glucose challenge) was comparable to the variation (5.5 %) for two non-fasting measurements [29]. Clinical and technical factors influencing SIF were investigated in 1185 T1DM patients. The results show that 33 % of the variance in SIF was explained by age, HbA1c, estimated glomerular filtration rate, smoking, skin tone, and clinic latitude [30]. Later, caffeine consumption was also found to contribute to SIF in the same cohort [31]. To the best of our knowledge, there are no reports concerning the influence of skin products on SIF.

## Skin fluorescence in diabetes screening

Skin fluorescence has been proposed to be useful as a cost-effective, simple, and reproducible test for diabetes screening [32]. In subjects with intermediate risk for developing T2DM, a SAF based decision tree was shown to have a similar or superior diagnostic performance for impaired glucose tolerance and diabetes in comparison with fasting plasma glucose, HbA1c, and the FINDRISC questionnaire [33]. The authors suggested that the decision tree could be used for selective or targeted screening for early diabetes in groups with increased risk. Cut-off values for SAF would be ≥80th (< 50 years) or ≥70th (≥ 50 years) age percentile, based on previously published reference values [21], for which patients should be referred for oral glucose tolerance tests. For the SCOUT device, a few studies have shown that SIF had a similar or superior performance to fasting plasma glucose, HbA1c, and/or capillary glucose in detecting abnormal glucose tolerance [29, 34, 35].

## Relations between SAF and AGE levels in different tissues

### Dissociation of SAF and plasma AGEs

In several studies, SAF levels are poorly related to simultaneously measured plasma or blood glycation markers [36–38]. The difference in turnover rate of skin and plasma proteins provides an obvious explanation. Accumulation of AGEs strongly depends on tissue turnover since AGEs are mainly irreversibly linked to tissue proteins. Turnover of collagen and elastin in the skin is very slow, with an estimated lifetime of dermal skin collagen of 10–15 years [39]. Therefore, dermal AGEs capture decades-long glycaemia. In contrast, AGEs derived from tissues with fast turnover (such as plasma, epidermis, and mucosa) are rapidly broken down to AGE peptides and/or free AGEs, which are excreted through the kidney. To illustrate, the conventional HbA1c glycation marker assay reflects a glycaemic period of 12 to 16 weeks. Some studies have shown that the poor relation of SAF with current HbA1c levels improves when a series of HbA1c levels, integrated over longer periods back in time (also mentioned as past long-term glycaemic control), are used [40–42].

### Associations between SAF and tissues with slow turnover

Examples of tissues with slow turnover are the eye lens, cartilage, cardiac tissue, and most brain cell/interstitial tissue types. Structural proteins in the eye lens remain lifelong present from 2–3 months after conception, while new proteins are only slowly added from the exterior part. Januszewski *et al*. [43] found that SAF and ocular (lens and cornea) fluorescence were elevated in T1DM compared to controls. Moreover SAF was correlated to lens- and cornea fluorescence in both non-diabetic and T1DM subjects. Hofmann *et al*. [44] reported a close correlation between SAF and tissue AGE levels of the atrial appendage. Both were associated with age and short- and long-term glucose levels in patients with coronary artery disease. These results provide evidence for a relation between SAF and cardiac tissue glycation.

## Micro - and macrovascular complications in diabetes

### AGE reader

Bos *et al*. [45] performed a systematic review of SAF and diabetes complications in 2011. The authors included studies in which patients participated with T1DM or T2DM, in which SAF levels were measured by the AGE Reader or its prototype (AFR), and in which information was drawn concerning complications of diabetes. In total, four cross-sectional studies [27, 46–48] and 3 prospective studies [49–51] met their inclusion criteria. In all seven studies, a positive association between SAF and one or more diabetes complications (all-cause mortality, cardiovascular mortality, micro- and macrovascular complications, neuropathy, and nephropathy) was seen, except for retinopathy. At that time, Bos *et al*. [45] concluded that these results should be interpreted with caution because studies were of large clinical heterogeneity, most studies included small numbers of subjects, and 5 of the 7 studies were from the same research group. Furthermore, all of these studies were performed in Europe, therefore, mainly including Caucasian subjects.

Recently, more studies confirmed the positive association between SAF and one or more complications. Four (cross-sectional) studies showed an independent relation between SAF and both micro- and macrovascular complications in T2DM [52–55]. Ahdi *et al*. [52] demonstrated that SAF was a stronger determinant for these complications than HbA1c in patients with fair skin colours, but not in patients with darker skin colour types (mainly Hindustani and Africans). Tanaka *et al*. [55] were first to describe that SAF was independently associated with the presence of micro- and macrovascular complications in a non-Caucasian (Japanese) population. Later, Liu *et al*. [53] confirmed this association in Chinese patients with diabetic foot ulcer. The authors found that SAF was independently associated with the occurrence of all the reported vascular diseases (retinopathy, nephropathy, peripheral neuropathy, coronary heart disease, cerebrovascular disease, peripheral artery disease), except for diabetic peripheral neuropathy. Finally, Noordzij *et al*. [54] showed that SAF was independently associated with the presence of both micro- and macrovascular complications in a multi-centre study performed in 5 Dutch hospitals.

Regarding diabetic retinopathy, two Japanese studies specifically investigated the relationship between SAF and the severity of the disease in T2DM [56, 57]. Both studies reported a positive association that remained significant after adjustment for (among others) age, HbA1c, diabetes duration, systolic blood pressure, and serum creatinine or presence of diabetic nephropathy. Moreover, the authors concluded that SAF was a better diagnostic marker for diabetic retinopathy compared to HbA1c. Hirano *et al*. [57] further reported that SAF was not correlated with diabetic macular oedema, neither was HbA1c or self-assessed diabetes duration.

In T1DM, four (cross-sectional) studies reported the relation of SAF with retinopathy, nephropathy, and/or neuropathy in the past 5 years [42, 58–60]. Except for one [58], all studies investigating retinopathy showed a significant correlation with SAF after correction for (at least) age [42, 59, 60]. With regard to nephropathy, all four studies showed some positive result. In one study [58], however, the positive association disappeared in multivariate analysis. Furthermore, it is important to mention that different definitions of the outcome have been used to detect nephropathy in these studies. The two studies investigating neuropathy both showed an independent relationship of SAF with neuropathy in multivariate analyses [58, 59].

In summary, these recent reports (performed by several research groups in different racial groups) show consistent evidence of an association between SAF and end-organ complications in diabetes. Several studies have now confirmed that SAF is associated with retinopathy, even after correction for age and nephropathy. In addition, a few studies established an independent association between SAF and vascular complications in diabetes patients of Asian descent. However, no relation was found between SAF and vascular complications in diabetes patients with darker skin colour types. Unfortunately, none of the recent studies had a prospective design, but from earlier studies, some evidence remains for the predictive value of SAF in the development of diabetes complications in T2DM [49, 50]. Therefore, prospective studies with long period of follow-up and large group size are still needed to establish the role and potential benefit of SAF in disease management of diabetes patients, especially in T1DM.

### SCOUT device

Three (cross-sectional) studies have been performed in which the association between SIF and diabetes complications is investigated [61–63]. All studies were performed in T1DM. First, Conway *et al*. [61] reported that SIF was more strongly associated with autonomic and distal symmetrical polyneuropathy compared to HbA1c. Later, Conway *et al*. [62] showed that SIF was significantly associated with coronary artery disease (CAD) after correction for age, diabetes duration, mean HbA1c, and sex. The authors concluded that SIF may be a useful overall marker of CAD risk in T1DM. Finally, Orchard *et al*. [63] investigated the association between SIF and complications in the DCCT/EDIC study. They found moderately strong associations with all complications. After adjustment for mean HbA1c, these associations remained significant for cardiac autonomic neuropathy, nephropathy, and coronary artery calcification in the conventional treatment group and became non-significant for all complications in the intensive treatment group. Orchard *et al*. [63] concluded that prospective studies are needed to fully evaluate the predictive value of SIF in the development of complications.

## SAF and clinical markers of cardiovascular disease

Supporting the association between SAF and cardiovascular complications, SAF has been related to several clinical markers of cardiovascular disease: diastolic function, arterial compliance, markers of endothelial function, and markers of atherosclerosis. A relation between SAF and functional anatomical changes, reflected in diastolic dysfunction, was first shown in a study by Hartog *et al*. [38] in patients with renal failure. Other studies have described the association between dermal or plasma AGEs and diastolic dysfunction [64, 65].

Several studies have shown that SAF is related to arterial compliance evaluated by arterial pulse wave velocity (aPWV). Ueno *et al*. [66] and Januszewski *et al*. [43] described a relation between SAF and aPWV in end stage renal failure and in diabetes patients, respectively. Similar relations were found in various patient groups by Watfa *et al*. [67], de Groot *et al*. [68], and Llaurado *et al*. [69]. In accordance with these studies, de Boer *et al*. [70] recently reported that SAF was associated with aPWV in recently diagnosed T2DM patients. In the same patient group, SAF was related to arterial calcification scores, although aPWV was not.

Relations between SAF and markers of microvascular and endothelial function have been described mainly in diabetes and renal failure patients. Araszkiewicz *et al*. [71] found a relation between SAF and microvascular reactivity, as assessed by peak flow after reactive hyperaemia measured by laser Doppler flow. Furthermore, Ueno *et al*. [72] observed a negative relation between SAF and circulating endothelial progenitor cells.

In earlier studies, it had already been shown that AGEs measured in skin samples using collagen fluorescence are associated with coronary calcium score, as a surrogate marker for coronary atherosclerosis. More recently, SAF has been reported to be associated with clinical markers of atherosclerosis, such as intima media thickness and coronary calcification score, by several groups [59, 73–75]. The relation of SAF and AGEs with calcification not only exists for coronary arteries, but also with medial artery calcification in peripheral arteries [76].

## Other prevalent conditions in diabetes

Vascular complications are common and receive much attention in diabetes research. More recently, diabetes have also been linked to other disorders, such as impaired cognitive function, Alzheimer’s disease [77], and depression [78]. Evidence exists that (small) vessel disease and reduced arterial compliance are linked to these complications. Recently, studies have also addressed the question whether SAF is linked to these disorders.

Spauwen *et al*. [79] reported inverse associations between SAF and cognitive performance. These associations were attenuated after adjustment for vascular risk factors and depression, but remained significant for delayed word recall and response inhibition and became non-significant for global cognitive functioning, immediate word recall, and information processing speed. These associations did not differ between individuals with and without T2DM. The authors concluded that AGEs may be involved in the development of cognitive decline, especially memory decline, partly through the action of vascular risk factors. Besides cognitive performance, SAF has also been associated with structural changes in the brain, namely lower grey matter volume [80]. Moran *et al*. [80] demonstrated that these results support further research into the role of AGEs in the pathogenesis of dementia in relation to T2DM.

Van Dooren *et al*. [81] showed that SAF is independently associated with presence of depressive disorders and symptoms of depression, both cognitive and somatic. These results did not differ between individuals with or without T2DM. The authors suggested that AGEs might be involved in the development of depression.

## Future perspectives

Diabetes has been shown to be a risk factor for retinal detachment after cataract surgery [82]. In a pilot study, our research group has recently shown that SAF was related to the severity of retinal detachment [83]. Soon, we will report the results of a prospective cohort study in which we investigated whether SAF and/or vitreous AGEs predict surgical failure in retinal detachment patients.

AGE measurements, SAF in particular, have been included in some large cohorts, such as the Maastricht study [84] (enriched with diabetes patients) and LifeLines [85]. These and other studies, such as the Derby CKD study [86–88], will provide more insight in the links between AGEs and other aging and disease mechanisms, especially when longer term follow-up data become available. Currently, our research group is investigating the performance of SAF in detecting abnormal glucose tolerance in a large population-based cohort study (LifeLines).

## Conclusion

It has been extensively shown that skin fluorescence is associated with a wide variety of long-term complications in diabetes mellitus. This association is supported by the relation between skin fluorescence and AGE accumulation in several tissues with slow turnover, between skin fluorescence and past long-term glycaemic control, and between skin fluorescence and clinical markers of cardiovascular disease. A limited number of studies have also provided evidence for an independent predictive role of skin fluorescence for cardiovascular complications and mortality in diabetes. More prospective studies, with longer follow-up period and larger group size, are being conducted to establish the predictive role and potential benefit of skin fluorescence in disease management of diabetes patients.
